# FBXL8 inhibits post-myocardial infarction cardiac fibrosis by targeting Snail1 for ubiquitin-proteasome degradation

**DOI:** 10.1038/s41419-024-06646-1

**Published:** 2024-04-13

**Authors:** Ya Li, Caojian Zuo, Xiaoyu Wu, Yu Ding, Yong Wei, Songwen Chen, Xiaofeng Lu, Juan Xu, Shaowen Liu, Genqing Zhou, Lidong Cai

**Affiliations:** 1 Department of Cardiology, Shanghai General Hospital, Shanghai Jiao Tong University School of Medicine, Shanghai, China; 2 Department of Key Laboratory, Lianshui County People’s Hospital, Kangda College of Nanjing Medical University, Huai’an, China

**Keywords:** Ubiquitylation, Translational research

## Abstract

Abnormal cardiac fibrosis is the main pathological change of post-myocardial infarction (MI) heart failure. Although the E3 ubiquitin ligase FBXL8 is a key regulator in the cell cycle, cell proliferation, and inflammation, its role in post-MI ventricular fibrosis and heart failure remains unknown. FBXL8 was primarily expressed in cardiac fibroblasts (CFs) and remarkably decreased in CFs treated by TGFβ and heart subjected to MI. The echocardiography and histology data suggested that adeno-associated viruses (AAV9)-mediated FBXL8 overexpression had improved cardiac function and ameliorated post-MI cardiac fibrosis. In vitro, FBXL8 overexpression prevented TGFβ-induced proliferation, migration, contraction, and collagen secretion in CFs, while knockdown of FBXL8 demonstrated opposite effects. Mechanistically, FBXL8 interacted with Snail1 to promote Snail1 degradation through the ubiquitin–proteasome system and decreased the activation of RhoA. Moreover, the FBXL8ΔC3 binding domain was indispensable for Snail1 interaction and degradation. Ectopic Snail1 expression partly abolished the effects mediated by FBXL8 overexpression in CFs treated by TGFβ. These results characterized the role of FBXL8 in regulating the ubiquitin-mediated degradation of Snail1 and revealed the underlying molecular mechanism of how MI up-regulated the myofibroblasts differentiation-inducer Snail1 and suggested that FBXL8 may be a potential curative target for improving post-MI cardiac function.

## Introduction

Although significant progress has been made in the therapy of coronary heart disease and acute myocardial infarction (MI) in the past two decades, MI is still the leading cause of heart failure [1, 2]. Cardiac fibroblasts (CFs) are the main cell type within the heart, which is the central cellular effector in cardiac fibrosis [3]. During the process of post-MI cardiac fibrosis, CFs are persistently differentiated into the phenotype of high secretory myofibroblasts [4], which express excessive extracellular matrix (ECM) and thus lead to increased wall stiffness, diastolic dysfunction, and heart failure. It is generally acknowledged that abnormal cardiac fibrosis is the dominant factor of pathological ventricular remodeling and heart failure post-MI [2]. Thus, exploring specific regulators for myofibroblast differentiation will provide promising treatments for post-MI cardiac fibrosis.

Snail1 is a zinc finger transcription factor, which is reported heavily implicated in epithelial-to-mesenchymal transition (EMT) and contributes to various pro-fibrotic diseases such as lung and liver fibrosis [5–8]. Recently, Clara et al. found that Snail1 was not normally expressed in adult fibroblasts but induced in fibroblasts undergoing wound repair [9]. High-expressed Snail1 in fibroblasts is involved in myofibroblast differentiation and fibrotic scar formation in infarcted hearts [10, 11]. In addition, research showed that the fibroblastic RhoA/αSMA pathway could be activated by TGFβ-induced Snail1 [12], which was proved to maintain myofibroblastic features, such as excessive fibronectin fibrillogenesis, collagen production, and formation of a rigid ECM. Therefore, Snail1 is a promising therapy target for myofibroblast differentiation.

The Snail1 protein is unstable and finely regulated by the ubiquitin–proteasome system. Several Skp1-Cullin1-F-box (SCF) E3 ligases have been reported to induce Snail1 ubiquitin-proteasome degradation [13, 14]. F-box proteins are substrate recognition of SCF E3 ubiquitin ligases that determine which proteins are ubiquitinated. The FBXL family represents the largest F-box proteins family which is characterized by a leucine-rich-repeat protein-binding domain [15]. Notably, the FBXL family is involved in various myocardial diseases, such as myocardial ischemia, reperfusion injury, and diabetic cardiomyopathy [16, 17]. In addition, hypoxia stabilizes Snail1 by down-regulating FBXL14 expression [14]. Among the FBXL family, a novel E3 ligase-FBXL8 has been reported to regulate cell proliferation and transformation by ubiquitin-dependent degradation of cyclin D3 in lymphomas [18]. However, the specific role of FBXL8 in post-MI cardiac fibrosis remains unclear.

Our study revealed that FBXL8 was predominantly expressed in CFs and hardly expressed in cardiomyocytes. FBXL8 overexpression inhibited myofibroblasts differentiation and ameliorated the process of post-MI ventricular fibrosis by targeting Snail1 for ubiquitin–proteasome degradation. These results characterized the anti-fibrosis ability of FBXL8 in cardiac fibrosis after MI.

## Methods

### Reagents

Antibodies against the following proteins were purchased from Proteintech: Snail1 (13099-1-AP, 1:1000 dilution); RhoA (10749-1-AP, 1:1000 dilution); α-SMA (14395-1-AP, 1:1000 dilution); Col1a1 (14695-1-AP, 1:1000 dilution); Flag (66008-4-Ig, 1:1000 dilution); HA (51064-2-AP, 1:1000 dilution); ubiquitin (10201-2-AP, 1:1000 dilution); GAPDH (60004-1-Ig, 1:1000 dilution); β-actin (81115-1-RR, 1:1000 dilution). GTP-RhoA antibody was purchased from NewEast Biosciences (26904, 1:1000 dilution). FBXL8 antibody was purchased from Santa Cruz (sc-390582, 1:1000 dilution). FBXL8 siRNA was purchased from Ribobio (Guangzhou, China). Adenoviruses overexpressing FBXL8 and green fluorescent protein (GFP) were purchased from Vigenebio (Rockville, MD, USA). CCG-1423 (HY-13991), Bafilomycin A1 (HY-100558), MG-132 (HY-13259), Cycloheximide (CHX, HY-12320), and TGFβ (HY-P70543) were purchased from MCE.

### Rat model of MI

Six-week-old male Sprague–Dawley rats (weight 200–250 g) were purchased from JieSiJie Laboratory Animal Co., Ltd. (Shanghai, China). All experimental rats were kept in standard cages (12-h light/dark cycle; temperature, 21 ± 1 °C; humidity, 55–60). Briefly, before grouping, each rat was allocated one of the consecutive random numbers from the random number table in order of body weight. Then, all rats were ranged by their random numbers from small to large, and every consecutive six rats were assigned as one group. We set the significance level (*α*) at 0.05 and power (1 − *β*) at 80% to determine the sample size according to our preliminary experiments. No samples or animals were excluded from the data analysis. The exact number of groups is included in figure legends. All experimental procedures were conducted in compliance with institutional animal care. The Shanghai General Hospital Institutional Animal Care and Use Committee has approved animal protocols in this study. Investigators were blinded to the allocation of different groups when performing outcome evaluations. We permanently ligated the left anterior descending (LAD) coronary artery to establish the MI model, as previously described [19]. Briefly, we anesthetized rats using 3% pentobarbital and conducted tracheal intubation and respiratory support for rats. Subsequently, we exposed the hearts of rats and ligated LAD with a 7-0 monofilament nylon suture, and the sham group received the same operation except for ligating the LAD.

### Echocardiography

The Vevo 770 system (VisualSonics, Toronto, Canada) was used to evaluate cardiac systolic function by two-dimensional and M-mode at 4 weeks after surgery. We measured the left ventricular (LV) internal dimension at end-systole (LVIDs) and LV internal dimension at end-diastole (LVIDd) by the LV parasternal long axis view. We figured out the ejection fraction (EF%) and fractional shortening (FS%) by the following formula: ${\rm{EF}} \% =[({{\rm{LVIDd}}}^{3}{\boldsymbol{-}}{{\rm{LVIDs}}}^{3})/{\rm{LVIDd}}^{3}]\times 100{;}{\rm{FS}} \% =[({\rm{LVIDd}}-{\rm{LVIDs}})/{\rm{LVIDd}}]\times 100$.

### Histology

Rat hearts were perfused and fixed with 4% paraformaldehyde for 24 h. Then, the hearts were cut horizontally, embedded into paraffin, and sliced for 5 µm thickness. The scar tissue area and infarct wall thickness of the cardiac sections were determined by Masson’s trichrome staining and assessed by Image J software.

### Fluorescent immunohistochemistry

The cardiac sections were deparaffinized, rehydrated and retrieved for antigen. The fibroblasts or cardiomyocytes were fixed by 4% formaldehyde and permeabilized by 0.1% Triton X-100. The cardiac sections or cells were blocked by 10% bovine serum albumin (BSA) for 15 min and incubated with primary antibodies at 4 °C overnight. The following primary antibodies were used for immunofluorescent staining: FBXL8 (sc-390582, 1:100 dilution), Snail1 (13099-1-AP, 1:200 dilution), α-SMA (14395-1-AP, 1:200 dilution), Col1a1 (14695-1-AP, 1:200 dilution). The next day, slides or cells were incubated for 90 min with Alexa Fluor 488 or 555-conjugated secondary antibodies and DAPI for 15 min at room temperature.

### Cell Culture and transfection

The human lung fibroblast (HLF) MRC-5 cell line was obtained from the American Type Culture Collection (ATCC) (Manassas, VA, USA), and the human dermal fibroblasts (HDF) CC-2511 cell line was obtained from Clonetics (San Diego, CA, USA). We isolated and cultured neonatal rat CFs from 1 to 2-day-old Sprague–Dawley rats, as previously described [19]. CFs underwent starvation in serum-free DMEM overnight and then stimulated with TGFβ (10 ng/ml) for 24 h. CFs were transfected with the indicated plasmids for 36 hours before TGFβ stimulation.

### Cell proliferation and migration assay

The ability of cell proliferation was evaluated by using a CCK-8 kit (MedChemExpress, HY-K0301) following the manufacturer’s protocols. We measured the absorbance of each well at 450 nm by using the microplate reader. Cell migration was determined by the transwell assay. Briefly, CFs were seeded in the upper chamber of 24-well plates in 100 µl serum-free culture medium incubated with or without TGFβ, and DMEM medium with 10% FBS was added into the lower chamber. The experimental group was transfected with FBXL8 plasmid or FBXL8 siRNA, and the control group was transfected with empty plasmid or control scramble. The next day, CFs were fixed by 4% formaldehyde, stained by hematoxylin and photoed.

### Cell contractile assay

In total, 100,000 CFs were seeded in collagen matrices and transferred to 24-well plates coated with 1% BSA. Serum-free DMEM with or without TGFβ was added in the culture well with the collagen gel releasing from the edges. Gels were scanned at the third day and ImageJ software was used to measure the surface diameter of the gels.

### Plasmid construction

The plasmids that encoded full-length human Fbxl8 with HA-tag HA-Fbxl8 aa 8–45 deleted mutant (8–45ΔF), HA-Fbxl8 aa 46–154 deleted mutant (46–154ΔC1), HA-Fbxl8 aa 155–254 deleted mutant (155–254ΔC2), HA-Fbxl8 aa 255–374 deleted mutant (255–374ΔC3), Flag-Snail1 and Flag-Snail1 fragments (C-terminal aa 152-264 and N-terminal aa 1–151) were purchased from WZ biosciences.

### Polymerase chain reaction

**Fig. 1:**
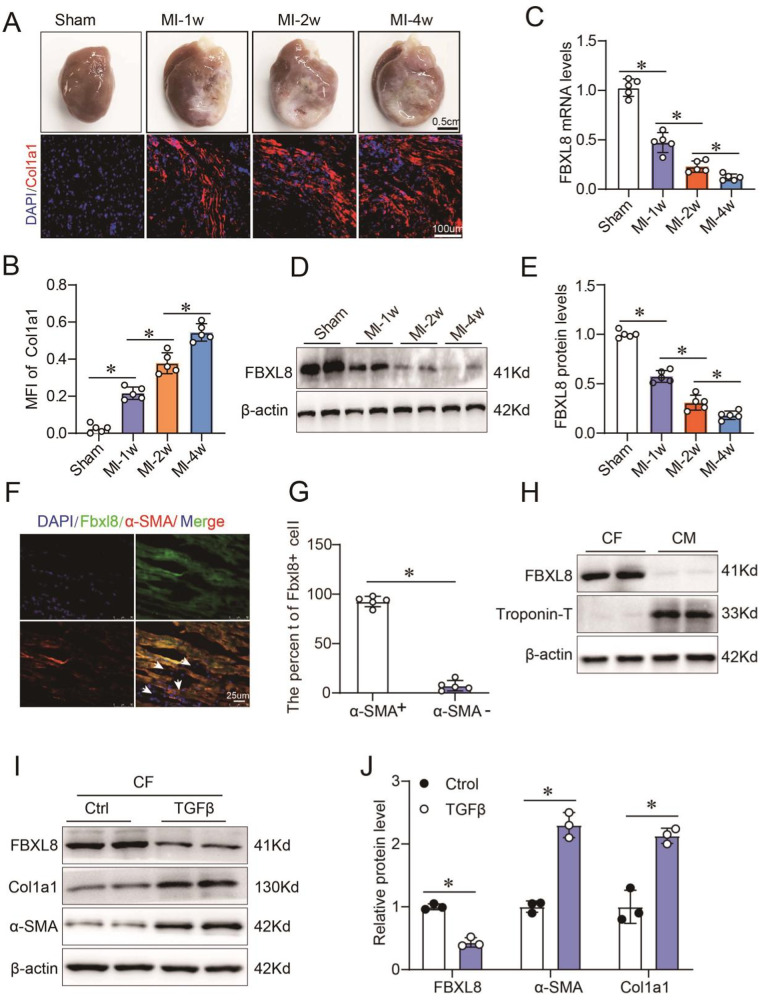
FBXL8 is primarily expressed in cardiac fibroblasts and remarkably downregulated in ischemic myocardium after MI. **A**, **B** The gross specimen, immunofluorescence (**A**), and quantitation (**B**) of Col1a1 in the myocardium of rats received sham or MI surgery at the indicated time points (*n* = 5). **C**–**E** the mRNA (**C**) and protein (**D**, **E**) of FBXL8 expression in hearts of sham and MI at 1 week (1w), 2w, and 4w after the operation (*n* = 5). **F**, **G** Immunofluorescence images (**F**) and quantitation (**G**) of LV-section staining from WT rats, showing FBXL8 expression (green) co-localization with α-SMA-positive cells (red). Scale bar: 25 μm. **H** Western blots of FBXL8 in cardiac fibroblasts (CFs) and cardiomyocytes (CMs). **I**, **J** Representative western blots (**I**) and quantitation (**J**) of FBXL8, α-SMA, and Cola1 protein levels in CFs treated with TGFβ (10 ng/ml) for 48 h (*n* = 3). Data are presented as mean ± SD. *P < 0.05.

We used Trizol reagent (Invitrogen) to extract total RNA following the product manual. The cDNA was synthesized by the PrimeScript™ RT reagent Kit (TAKARA), and we used SYBR green PCR master mix (Roche) to perform qPCR following the product manual. The FBXL8 mRNA level in Fig. 1C was normalized by the mRNA of β-actin expression. Relative expression calculated by means of 2^−^^ΔΔCT^. PCR products were run on agarose gel and then separated on 6% non-denaturing polyacrylamide gel. Quantitation of band density was performed using ImageJ software. The Snail1 mRNA in Fig. 5A was normalized by the mRNA of GAPDH expression. The supplementary Table 1 showed the primer pairs used in this study.

### Western blot

We used the RIPA buffer (Beyotime Biotechnology) to lyse the ventricular tissue and cells. The supernatant was extracted after tissue or cell lysates were centrifuged at 12,000 × *g* for 10 min at 4 °C. SDS-PAGE gel was used to separate the protein extractions, and then the separated protein was transferred to the PVDF membranes. The PVDF membranes were incubated with indicated primary antibodies overnight at 4 °C after blocking with 5% milk for 1 h at room temperature. On the second day, the PVDF membranes were washed by TBST for 10 min and incubated with the secondary antibodies for 60 min at room temperature. The Tanon 5200 was used to capture the protein bands. We used β-actin or GAPDH to normalize the specific protein levels.

### Immunoprecipitation (IP) assays

All IP tests were performed by using commercially available kits (#635696; Takara). Cultured HEK293T cells were transiently transfected with HA-FBXL8 and Flag-Snail1 plasmids and then cultured for 48 h. Then, the cells were lysed in an ice-cold IP buffer containing a protease inhibitor cocktail and centrifuged at 13,000×*g* for 15 min. The obtained cell lysates were precleared with Protein A/G-agarose beads for 3 h and then incubated with the indicated antibody at 4 °C overnight. The immune complex was collected after washing with cold IP buffer and subjected to immunoblot using the indicated primary antibodies and the corresponding secondary antibodies.

### Ubiquitination assays

Cultured CFs were lysed in SDS lysis buffer (20 mM Tris-HCl, pH 7.4, 150 mM NaCl, 1 mM EDTA, and 1% SDS) containing protease inhibitors (Beyotime, P1010). The lysates were denatured by heating for 5 min at 95 °C, diluted 10-fold by lysis buffer, and centrifuged at 20,000×*g* at 4 °C for 30 min. The supernatant was immunoprecipitated with the indicated antibodies, followed by a Western blot.

### Statistical analysis

Data are presented as mean ± SD. The SPSS version 16.0 was used to analyze data. The Shapiro–Wilk test was used to evaluate the Gaussian distribution of the data. An unpaired, two-tailed Student *t*-test was used in comparisons between the two groups. One-way ANOVA followed by the post hoc Tukey test was used to analyze multi-group comparisons. *P* < 0.05 was considered a statistically significant difference.

## Results

### FBXL8 is downregulated in the cardiac tissue of post-MI rats and mainly expressed in CFs

To evaluate the function of FBXL8 in post-MI, we first examined the expression of FBXL8 in cardiac tissues after MI at the indicated time. Gross morphology of the heart and increased Col1a1 concentration in the border zone of MI showed the development and progression of pathological cardiac remodeling post-MI over time (Fig. 1A, B). Intriguingly, the mRNA and protein of FBXL8 expression were down-regulated in the infarcted myocardium compared with sham-operated rats (Fig. 1C–E). We further observed that FBXL8 was selectively enriched in CFs, as the IF staining of cardiac tissue from normal rats indicated that FBXL8 staining is highly co-localized with α-SMA (Fig. 1F, G). In addition, IF staining showed that FBXL8 is enriched in CFs, whereas hardly detected in cardiomyocytes (CMs) (Fig. S1A, B). Consistently, the western blot indicated the FBXL8 protein expression in CFs was significantly higher than that in CMs (Fig. 1H). Next, we further assessed the FBXL8 expression in the differentiation of myofibroblasts. TGFβ, as the well-known pro-fibrotic factor, was used to establish an in vitro cardiac fibrosis model. The protein levels of Col1a1 and α-SMA increased significantly in TGFβ-treated CFs, indicating that we successfully established the fibrotic cellular model. Western blotting revealed that FBXL8 protein expression was significantly down-regulated in TGFβ-treated CFs compared with the control group (Fig. 1I, J). The decreased expression of FBXL8 in the myocardium post-MI and CFs differentiation indicates an important role of FBXL8 in post-MI fibrosis.

### FBXL8 prevents TGFβ-induced myofibroblast differentiation in vitro

**Fig. 2:**
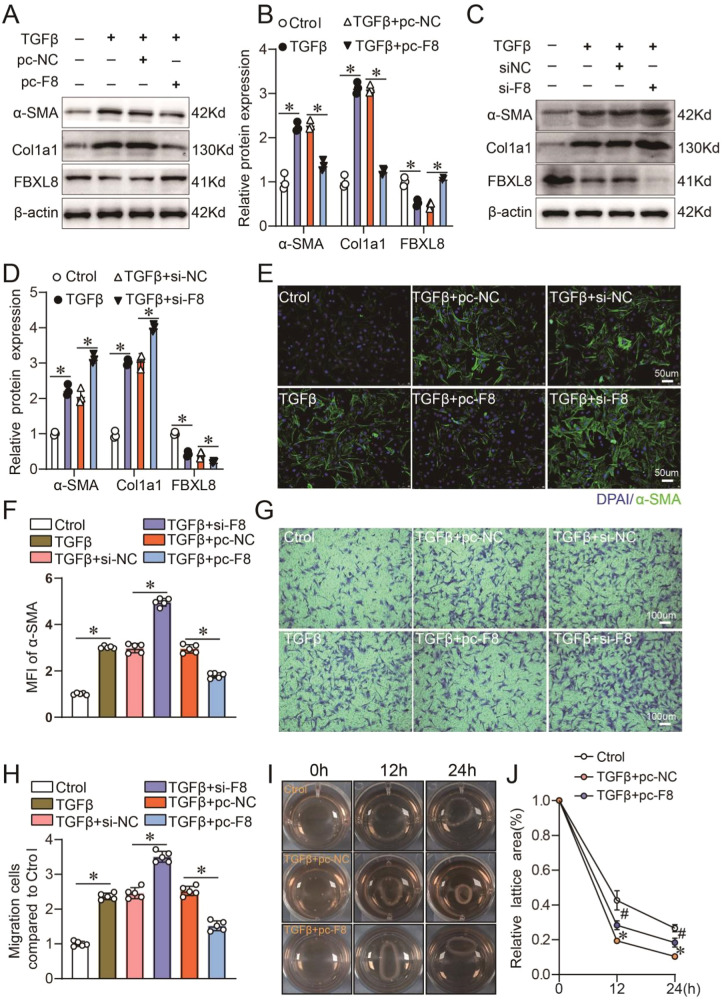
FBXL8 regulates TGFβ-induced myofibroblast differentiation. **A**, **B** Representative western blots (**A**) and quantification (**B**) of α-SMA, Col1a1, and FBXL8 protein levels in CFs treated with control or FBXL8 plasmid, and then treated with TGFβ stimulation for 48 h (*n* = 3). ^*^P < 0.05. **C**, **D** Representative western blots (**C**) and quantification (**D**) of α-SMA, Col1a1, and FBXL8 protein levels in CFs treated with negative control or FBXL8 siRNA, and then treated with TGFβ stimulation for 48 h (*n* = 3). ^*^P < 0.05. **E**, **F** CFs were either treated with vectors, FBXL8 plasmid, or FBXL8 siRNA and then treated with TGFβ stimulation for 48 h. Immunofluorescence staining of α-SMA in CFs (**E**) was performed for each group, and then the mean fluorescent intensity (**F**) of each group was calculated and analyzed (*n* = 5). α-SMA: Green, magnification: 200×, Scale bar = 50 μm. ^*^P < 0.05. **G**, **H** Transwell assay was used to detect the migration ability of each group (*n* = 5). ^*^P < 0.05. **I**, **J** Representative images (**I**) and quantification (**J**) of gel contraction in the indicated groups (*n* = 5). ^*^P < 0.05 vs. Ctrol; ^#^*P* < 0.05 vs. TGFβ + pc^−^NC. Data are presented as mean ± SD.

To elucidate the roles of FBXL8 in the phenotype of cardiac fibroblasts, we used siRNA and plasmids to manipulate the expression of FBXL8 in CFs. We found that the pro-fibrotic effects of TGFβ were largely regulated by FBXL8, illustrated as the upregulation expression of α-SMA and Col1a1 induced by TGFβ were significantly inhibited by FBXL8 overexpression (Fig. 2A, B). Reciprocally, FBXL8 knockdown remarkably promoted TGFβ induced pro-fibrotic protein changes in CFs (Fig. 2C, D), indicating that FBXL8 prevented TGFβ-induced myofibroblast differentiation. To further provide evidence of FBXL8 in the regulation of myofibroblast differentiation, immunofluorescence analysis was performed. The results of α-SMA staining showed similar results, that FBXL8 knockdown increased the mean fluorescence intensity (MFI) of α-SMA by 1.6-fold (Fig. 2E, F). FBXL8 overexpression significantly decreased the MFI of α-SMA induced by TGFβ in CFs (Fig. 2E, F). Next, we assessed the pro-fibrotic hallmarks of activated CFs, i.e., migration, proliferation, and the acquired ability to contract ECM. These results indicated that FBXL8 knockdown facilitated TGFβ-induced CFs migration and proliferation by 1.4- and 1.3-fold, respectively (Fig. 2G, H and Fig. S2). In contrast, FBXL8 overexpression hampered the capabilities of migration and proliferation upregulated by TGFβ stimulation (Fig. 2G, H and Fig. S2). Most significantly, FBXL8 overexpression reduced the contraction ability of CFs (Fig. 2I, J). Therefore, FBXL8 prevented TGFβ-dependent myofibroblast differentiation.

### RhoA/α-SMA signaling is involved in FBXL8-regulated myofibroblast differentiation

**Fig. 3:**
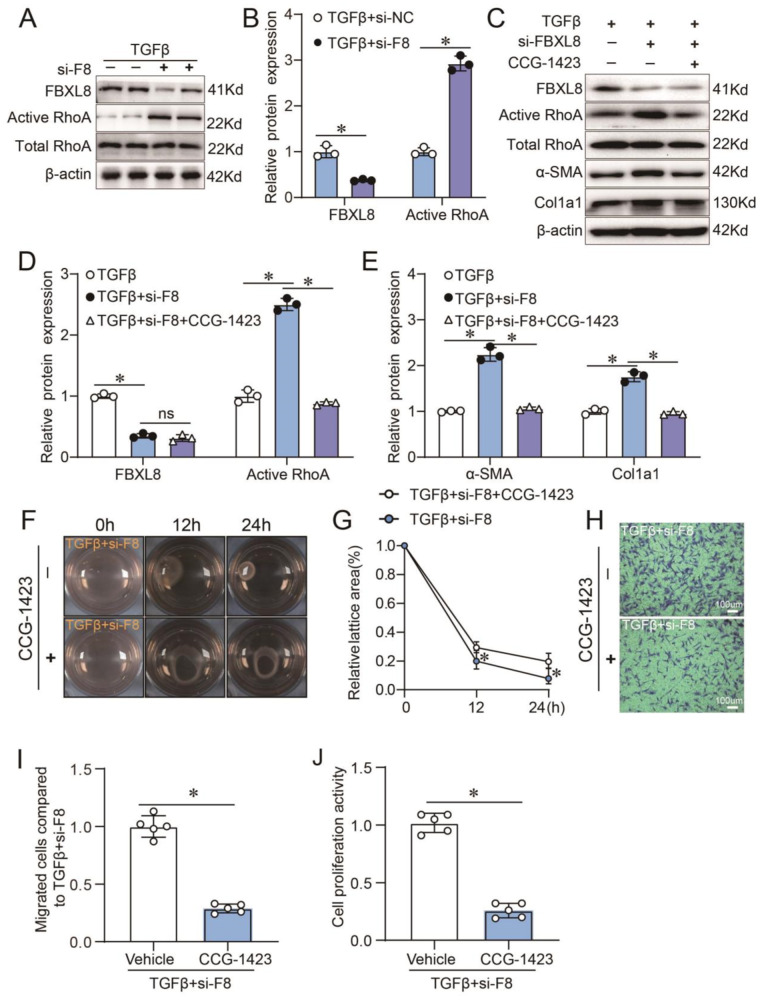
RhoA/α-SMA signaling is involved in FBXL8-regulated myofibroblast differentiation. **A**, **B** Immunoblotting (**A**) and quantitation (**B**) of FBXL8, active RhoA, and total RhoA protein levels in cells transfected with negative control or FBXL8 siRNA and then treated with TGFβ (10 ng/ml) for 36 h (*n* = 3). **C**–**E** Immunoblotting (**C**) and quantitation (**D**, **E**) of FBXL8, active RhoA, total RhoA, α-SMA, and Col1a1 protein levels in the cells transfected with FBXL8 siRNA with or without CCG-1423 (10 μM) and then treated with TGFβ (10 ng/ml) for 36 h (*n* = 3). **F**, **G** Representative image (**F**) and quantification (**G**) of gel contraction in the indicated groups (*n* = 5). **H**, **I** Representative transwell images (**H**) and quantification (**I**) of migrated cells in the indicated groups (*n* = 5). **J** Quantification of cell proliferation in the indicated groups by CCK8 assay (*n* = 5). Data are presented as mean ± SD. ^*^P < 0.05.

RhoA is a member of the Ras superfamily of GTP-binding proteins. There is growing evidence that the Rho GTPase activity is indispensable for α-SMA synthesis and stress fiber formation [20, 21]. Considering the essential role of FBXL8 in the regulation of TGFβ-induced myofibroblast differentiation, we then tested the influence of FBXL8 on the activities of Rho GTPase. We found that the active RhoA-GTP is remarkably upregulated in FBXL8-knockdown CFs by 2.9 fold (Fig. 3A, B). Next, to confirm the role of RhoA in FBXL8-regulated myofibroblast differentiation, we applied a chemical agent, CCG-1423 (10 μM), which is a small-molecule inhibitor of RhoA transcriptional signaling. Interestingly, we found that the application of CCG-1423 almost completely abolished the amplifying effects of FBXL8 knockdown on myofibroblast differentiation induced by TGFβ, demonstrated as the upregulation expression of α-SMA and Col1a1 in FBXL8 knockdown CFs induced by TGFβ were completely inhibited by CCG-1423 (Fig. 3C–E). In line with the changes of fibrotic markers, the in vitro migration, proliferation, and collagen gel contractile abilities induced by TGFβ in FBXL8 knockdown CFs were also remarkably prevented by CCG-1423 (Fig. 3F–J). Therefore, FBXL8 inhibited myofibroblast differentiation by inactivating RhoA/α-SMA signaling.

### The ΔC3 domain of FBXL8 binds with the C-terminal domain of Snail1

**Fig. 4:**
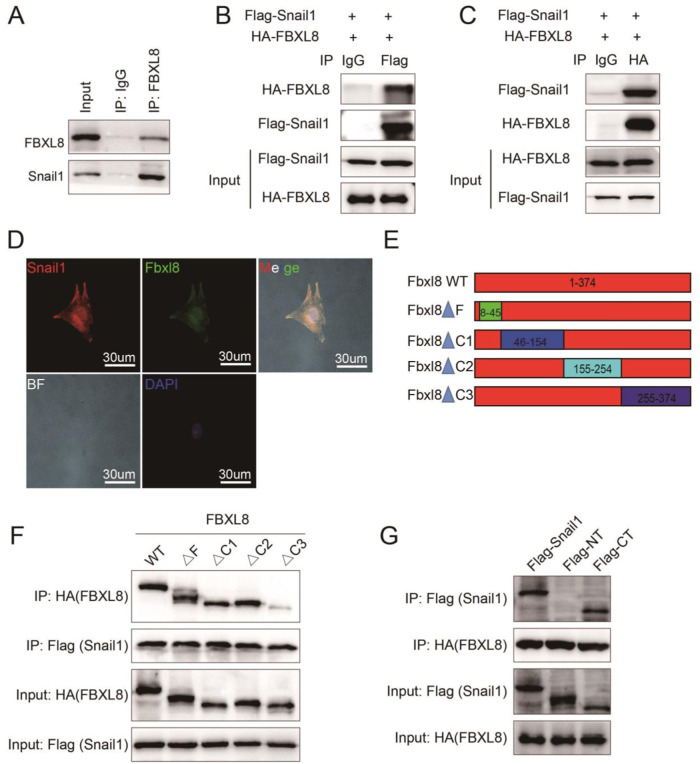
The ΔC3 domain of FBXL8 interacts with the C-terminal domain of Snail1. **A** Co-IP assay of endogenous FBXL8 and Snail1 in rat CFs. **B**, **C** Co-IP assays of the exogenous interaction between HA-FBXL8 and Flag-Snail1 in HEK293T cells transfected with the indicated plasmids. **D** Representative confocal images of the colocalization of FBXL8 and Snail1 in CFs treated with TGFβ. **E** Schematic model of deletion mutants of FBXL8. **F** Co-IP analysis of the binding regions of FBXL8 and Snail1. **G** Co-IP analysis of the binding regions of Snail1 and FBXL8.

To explore the mechanism by which FBXL8 regulates myofibroblast differentiation, we took the intersection between the 10 potential binding proteins from BioGRID and the 1801 MI-related proteins from DisGeNet, a database of human gene-disease associations. Snail1 and HSP90AA1 were the 2 potential binding proteins related to MI (Fig. S3). Among them, Snail1 is the well-recognized transcription factor in TGFβ signaling that regulates myofibroblast differentiation [11]. To investigate the combination between FBXL8 and Snail1, we performed exogenous and endogenous co-immunoprecipitation (co-IP) assays. The endogenous co-IP assay showed FBXL8 bound with Snail1 in CFs (Fig. 4A). As shown in Fig. 4B, HA-FBXL8 was immunoprecipitated by the anti-Flag antibody specifically rather than by control IgG (Fig. 4B) and Flag-Snail1 was immunoprecipitated by anti-HA antibody but not IgG antibody (Fig. 4C). We further investigated the localization of FBXL8 and Snail1 in primary CFs. Immunostaining showed that FBXL8 and Snail1 were co-localized in both the cytosol and the nucleus (Fig. 4D). To confirm the domain of FBXL8 interaction with Snail1, we generated the FBXL8 deletion mutants (Fig. 4E). NIH3T3 cells were transfected by HA-FBXL8 (wild type, ΔF, ΔC1, ΔC2, and ΔC3) or HA-GFP along with Flag-Snail1. We found the interaction between Snail1 and FBXL8ΔC3 was reduced (Fig. 4F). To identify the interaction region of FBXL8 with Snail1 protein, Flag-Snail1 deletion mutants comprising the C-terminal (CT) (Flag-CT: amino acids 152-264) or the N-terminal (NT) (Flag-NT: 1–151) parts of Snail1 were generated. As shown in Fig. 4G, FBXL8 is bound with the the CT domain of Snail1. Thus, the ΔC3 domain of FBXL8 directly binds with the CT domain of Snail1.

### FBXL8-mediated ubiquitination triggers the proteasome degradation of Snail1

**Fig. 5:**
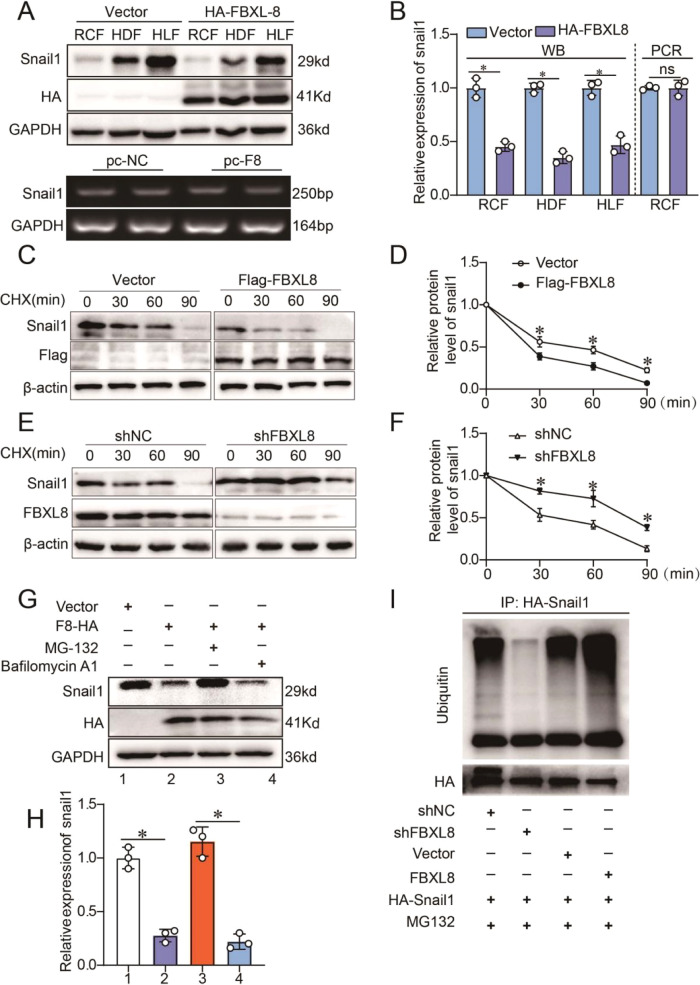
FBXL8 targets Snail1 for ubiquitin-proteasome degradation. **A**, **B** The mRNA, protein level (**A**), and quantification (**B**) of Snail1 mediated by FBXL8 overexpression (*n* = 3). **C**–**F** CFs transfected with Flag-vector, Flag-FBXL8 plasmids (**C**, **D**), control shRNA, or FBXL8 shRNA (**E**, **F**) were treated with 40 μg/mL CHX for the indicated periods and then analyzed by Western blotting (n = 3). **G**, **H** Western blotting (**G**) and quantification (**H**) of Snail1 in CFs following HA-FBXL8 overexpression for 24 h and stimulated with TGFβ for another 36 h, MG-132 (50 μM) or Bafilomyocin A1 (50 μM) for 6 h (*n* = 3). **I** Western blot of ubiquitination assays assessing the ubiquitin level of Snail1 after FBXL8 overexpression or knockdown for 24 h and treated with MG-132 (50 μM) for 6 h in HEK293T cells. RCF rat cardiac fibroblast, HDF human dermal fibroblast, HLF human lung fibroblast. Data are presented as mean ± SD. ^*^P < 0.05.

Next, we explored the mechanism of FBXL8 in regulating the Snail1 expression. Intriguing, we found that FBXL8 overexpression had no influence on the expression of Snail1 in mRNA level but remarkably decreased the expression of Snail1 protein (Fig. 5A, B), indicating that FBXL8 may regulate the Snail1 protein level by post-translational modifications. To provide additional evidence of FBXL8 in the modulation of the stability of Snail1 protein, we applied cycloheximide (CHX), a translation inhibitor, to exclude the impacts of Snail1 mRNA translation. We overexpressed FBXL8 and examined Snail1 protein stability after CHX treatment. Western blotting revealed that FBXL8 overexpression shortened the half-life of endogenous Snail1 (Fig. 5C, D), whereas FBXL8 knockdown prolonged the half-life of the Snail1 protein (Fig. 5E, F). Next, MG-132 and Bafilomycin A1 were used to explore the molecular mechanisms of how FBXL8 regulates the Snail1 protein level. We found that Snail1 down-regulation mediated by FBXL8 overexpression was inhibited by the proteasome inhibitor MG-132 but not by lysosomal inhibitor Bafilomycin A1 (Fig. 5G, H). To further examine whether Snail1 is a ubiquitination substrate of FBXL8, we performed an in vitro ubiquitination assay and showed that FBXL8 ligase could poly-ubiquitinate Snail1 in cells (Fig. 5I). Thus, FBXL8 targets Snail1 for ubiquitin-proteasome degradation.

### FBXL8-regulated myofibroblast differentiation is dependent on Snail1

**Fig. 6:**
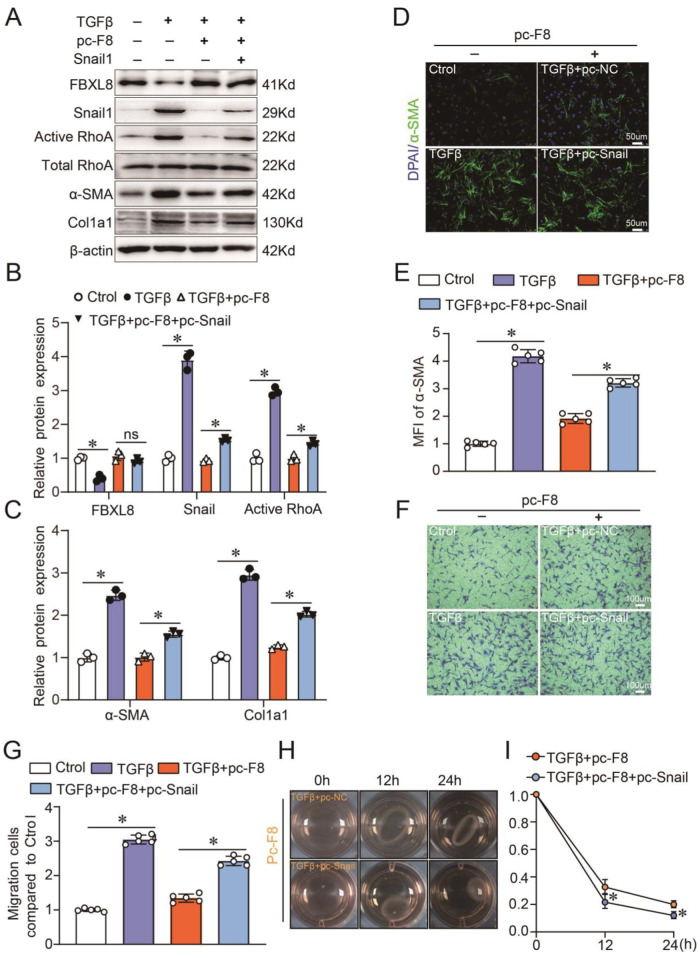
Snail1 supplement abolishes FBXL8-regulated myofibroblast differentiation. **A**–**C** Representative western blots (**A**) and quantification (**B**, **C**) of α-SMA, Col1a1, active RhoA, total RhoA, FBXL8, and Snail1 protein levels in cardiac fibroblasts transfected with FBXL8 or FBXL8 + Snail1 for 24 h and then stimulated by TGFβ (10 ng/ml) for another 36 h (*n* = 3). **D**, **E** Immunofluorescence (**D**) and quantitation (**E**) of α-SMA in CFs (*n* = 5). **F**, **G** Representative image (**F**) and quantification (**G**) of migrated cells in the indicated groups (*n* = 5). **H**, **I** Representative image (**H**) and quantification (**I**) of gel contraction in the indicated groups (*n* = 5). Data are presented as mean ± SD. ^*^P < 0.05.

Snail1/RhoA/α-SMA pathway is a proven mechanism involved in fibroblast activation [12], and several studies have demonstrated that Snail1 participated in organ fibrosis, i.e., renal fibrosis, cardiac fibrosis, and lung fibrosis [10, 22, 23]. On the basis of these observations, the role of Snail1 in FBXL8-regulated myofibroblast differentiation is to be investigated. We co-transfected CFs with FBXL8 or Snail1 plasmids for 24 h and then stimulated by TGFβ for another 36 h. As shown in Fig. 6A–C, FBXL8 overexpression inhibited the expression of α-SMA, Col1a1, as well as active RhoA induced by TGFβ in CFs, whereas Snail1 overexpression reversed this inhibitory effect of FBXL8 overexpression. Expression of Snail1 in fibroblasts is necessary for the activation of RhoA and the acquisition of a myofibroblastic phenotype [12]. Thus, FBXL8 regulates the RhoA activity and myofibroblastic phenotype by targeting Snail1 for ubiquitin-proteasome degradation. Consistent with the western blot results, immunofluorescence of α-SMA showed similar results, that FBXL8 overexpression decreased the MFI of α-SMA in CFs treated by TGFβ and Snail1 overexpression significantly reversed the inhibitory effect of FBXL8 overexpression (Fig. 6D, E). Furthermore, transwell and collagen gel contractile assays were conducted to measure cell migration and contraction capability. In line with the above-mentioned results, the migration and contraction abilities upregulated by TGFβ stimulation were remarkably hampered by FBXL8 overexpression, which was partially restored by Snail1 overexpression (Fig. 6F–I). In conclusion, Snail1/RhoA/α-SMA pathway is implicated in FBXL8-regulated myofibroblast differentiation.

### FBXL8 overexpression attenuates MI-induced cardiac dysfunction and fibrosis

**Fig. 7:**
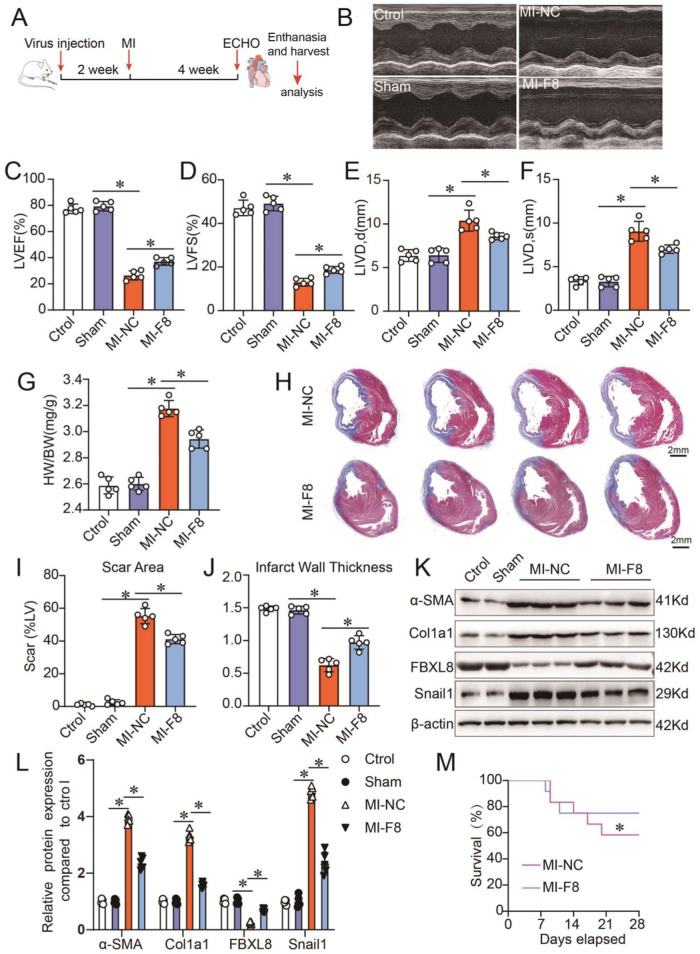
Gene therapy of FBXL8 improves cardiac function and inhibits cardiac fibrosis after MI. **A** Schematic diagram of the experimental set-up. **B** Representative echocardiographic images 4 weeks after MI. **C-F** Measurement of cardiac function indexes EF (**C**) and FS (**D**) as well as the left ventricular dilatation indexes LVIDd (**E**) and LVIDs (**F**) from echocardiographic results as in (**B**) (*n* = 5). **G** Heart weight/body weight ratios (HW/BW) from each group (*n* = 5). **H**–**J** Masson staining of infarcted heart sections (**H**) and quantitative analysis of the scar area (**I**) and wall thickness (**J**) of the infarct regions from WT and FBXL8 overexpression hearts at day 28 after MI (*n* = 5). Scale bars: 2 mm. **K**, **L** Representative western blots (**K**) and quantification (**L**) of α-SMA, Col1a1, Snail1, and FBXL8 at day 28 post-MI in the infarct area for each group (*n* = 5). **M** Kaplan–Meier survival analysis post-MI in MI-NC (*n* = 11) and MI-FBXL8 rats (*n* = 12). Data are presented as mean ± SD. ^*^P < 0.05. LVEF left ventricular ejection fraction, LVFS left ventricular fractional shortening, LVIDd left ventricular internal diameter diastolic, LVIDs left ventricular internal diameter diastolic.

To further confirm the role of FBXL8 in cardiac fibrosis post-MI, we generated AAV9 viruses encoding an open reading frame of rat FBXL8. Particles of AAV-9 (5 × 10^11^ GC) were injected in the hearts of 8-week-old rats 2 weeks before MI or sham surgery, and echocardiography analysis was performed after 4 weeks post-MI (Fig. 7A). We found that FBXL8 overexpression (MI-F8) significantly improved cardiac function post-MI, demonstrated as a higher left ventricular ejection fraction (LVEF) and Fractional shortening (FS) and a smaller left ventricular internal diameter diastolic (LVIDd) and left ventricular internal diameter systolic(LVIDs) (Fig. 7B–F). In parallel, with FBXL8 gene therapy, the heart weight/body weight (HW/BW) ratio was remarkably lower than in MI-NC rats (Fig. 7G). Masson’s trichrome-stained LV sections showed that myocardial fibrosis induced by MI was dramatically attenuated in FBXL8 overexpression rats compared with MI-NC rats, exhibited a smaller scar area and thicker infarcted wall thickness (Fig. 7H–J). Besides, the protein levels of fibrosis-related genes, including α-SMA and Col1a1 were markedly decreased in the myocardial tissues of MI-F8 rats (Fig. 7K, L). Mechanistically, FBXL8 increased the ubiquitination level of Snail1 (Fig. S4) and decreased Snail1 expression (Fig. 7K, L). Consistently, the post-MI survival rate of FBXL8 overexpression rats was higher than that of MI-NC rats (Fig. 7M). In conclusion, FBXL8 gene therapy protects the heart against MI-induced pathological remodeling and cardiac dysfunction.

## Discussion

**Fig. 8:**
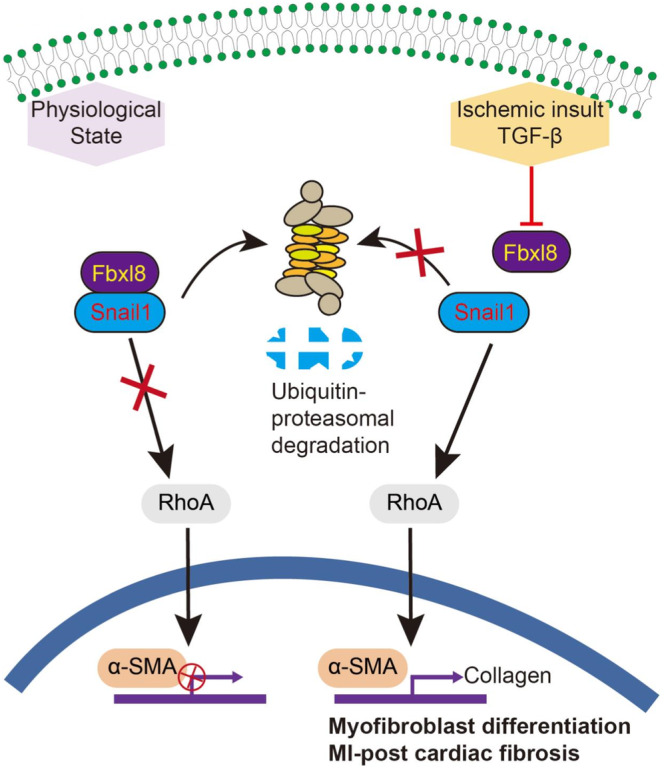
Schematic of the proposed model for the FBXL8-mediated proteasome degradation of Snail1 in cardiac fibrosis. FBXL8 interacts with Snail1 and targets Snail1 for ubiquitin-proteasome degradation response to MI. In response to TGFβ stimulation and MI, downregulation of FBXL8 expression in CFs leads to stabilization of Snail1, thereby activating the RhoA/α-SMA pathway, inducing myofibroblast differentiation and the progress of cardiac fibrosis following MI.

Cardiac fibroblast differentiation into hyper-secretory, hyper-proliferative myofibroblasts is the key event in pathological cardiac fibrosis post-MI, which is the main cause leading to the development of cardiac dysfunction and arrhythmia [24, 25]. Identification of underlying molecular targets of this process is important for improving preventive and therapeutic strategies. Our present work demonstrated that FBXL8 was predominantly expressed in CFs, which was remarkably decreased in CFs upon TGFβ stimulation and post-MI myocardium tissue. FBXL8 overexpression could inhibit the differentiation of CFs into myofibroblasts induced by TGFβ. In addition, FBXL8 overexpression significantly alleviated the pathological cardiac remodeling in response to MI injury with improved cardiac function and attenuated cardiac fibrosis. Mechanistically, the FBXL8-Snail1/RhoA/α-SMA pathway was identified to regulate CFs activation (Fig. 8). Therefore, FBXL8 plays a crucial role in cardiac fibrosis and could be a potential therapeutic strategy for pro-fibrotic cardiac remodeling in heart failure patients.

Cumulative studies have demonstrated that the SCF E3 ubiquitin ligase family acts as an important regulator of post-translational modification and is closely associated with cardiovascular diseases such as cardiac hypertrophy, myocardial infarction, arrhythmia, and myocardiotoxicity [26, 27]. For example, FBXL22 was identified as a cardiac-enriched F-box protein that modulates sarcomeric protein turnover and is critical for maintaining contractile function in vivo [28]. F-box and WD repeat domain-containing 7 (FBXW7) were proven to regulate cardiac hypertrophy through the EZH2–SIX1 axis [29]. FBXW5 negatively regulates pathological cardiac hypertrophy by inhibiting the TAK1 signaling pathway [30]. In this study, we proved for the first time that FBXL8 exerted a cardiac protective role in the heart, as our experimental data demonstrated that FBXL8 overexpression significantly alleviated pathological cardiac remodeling after MI, improved cardiac function, and attenuated fibrosis.

Cardiac fibrosis is the hallmark of various heart diseases and a primary factor of pathological ventricular remodeling and progressive deterioration of post-MI LV function [31]. However, there are limited therapeutic approaches to effectively reduce or reverse cardiac fibrosis. Myofibroblasts are the central cellular effector in the production of collagen, which is primarily derived from cardiac resident fibroblasts [32]. Therefore, prevention of the differentiation of fibroblasts to myofibroblasts is important in inhibiting pathological cardiac fibrosis. In our present study, we found that FBXL8 was predominantly expressed in CFs and remarkably decreased in CFs treated by TGFβ. FBXL8 overexpression prevented myofibroblast differentiation and ECM expression induced by TGFβ, demonstrated by decreased expression of the fibrosis-associated protein (α-SMA) and Cola1 and the capacity of proliferation, migration, and contraction of CFs in vitro. Conversely, FBXL8 knockdown in CFs promoted TGFβ-stimulated myofibroblast differentiation, proliferation, migration, and contraction in vitro. Moreover, gene therapy of FBXL8 overexpression targeting CFs significantly attenuated myocardial fibrosis after MI. These results indicated that FBXL8 could be a potential therapeutic target for pro-fibrotic cardiac remodeling in heart failure patients.

Another interesting finding of our study is that we proved FBXL8 is a ubiquitin ligase controlling Snail1 protein stability. Snail1 is a zinc finger transcription factor, which is not normally expressed in adult fibroblasts, but highly expressed in myofibroblasts [11]. Substantial evidence has proved that Snail1 plays a crucial role in EMT and is associated with organ fibrosis, such as lung and renal fibrosis [22, 23]. Recently, Stanisavljevic et al. provided additional insight into the role of Snail1 in CFs activation, and Snail1-deleted fibroblast failed to gain myofibroblast characteristics, such as α-SMA expression and collagen secretion [12]. Previous studies reported that F-box proteins, such as FBXL5, FBXL7, and FBXL14, targeted Snail1 for ubiquitin–proteasome degradation, indicating that the F-box family owns a common motif involved in the regulation of Snail1 ubiquitin-proteasome degradation [13, 14, 33]. It is well recognized that RhoA activation is required for stress fiber formation and α-SMA expression in differentiated CFs [20, 21]. Consistently, we found that FBXL8 overexpression significantly inhibited RhoA activation induced by TGFβ, and the RhoA inhibitor abolished the FBXL8 knockdown-induced α-SMA expression and collagen production. Thus, we speculated that FBXL8 mediated its cardiac-protective role in MI by Snail1/RhoA/α-SMA pathway.

In conclusion, our study proved that the FBXL8 exerted a cardiac protective post-MI, and FBXL8 markedly inhibited myofibroblasts differentiation and restrained the progress of post-MI cardiac fibrosis by targeting Snail1 for ubiquitin–proteasome degradation. Additionally, the Snail1/RhoA/α-SMA pathway was confirmed to mediate CFs activation and cardiac fibrosis. The interaction of FBXL8-Snail1 in the heart may provide a promising therapy for preventing cardiac fibrosis and heart failure after MI.

### Supplementary information

Supplementary figures

Western blot original data

## Data Availability

All data generated during this study are included in this published article and its supplementary information files.
