# Supplementary material for: FBXL8 inhibits post-myocardial infarction cardiac fibrosis by targeting Snail1 for ubiquitin-proteasome degradation
Source: Cell Death Dis. 2024 Apr 13;15(4):263. doi: 10.1038/s41419-024-06646-1 (PMC11016067; doi:10.1038/s41419-024-06646-1)
Supplement: Supplementary file 1 — Supplementary figures [file 41419_2024_6646_MOESM1_ESM.docx]

**Supplementary figures**

**
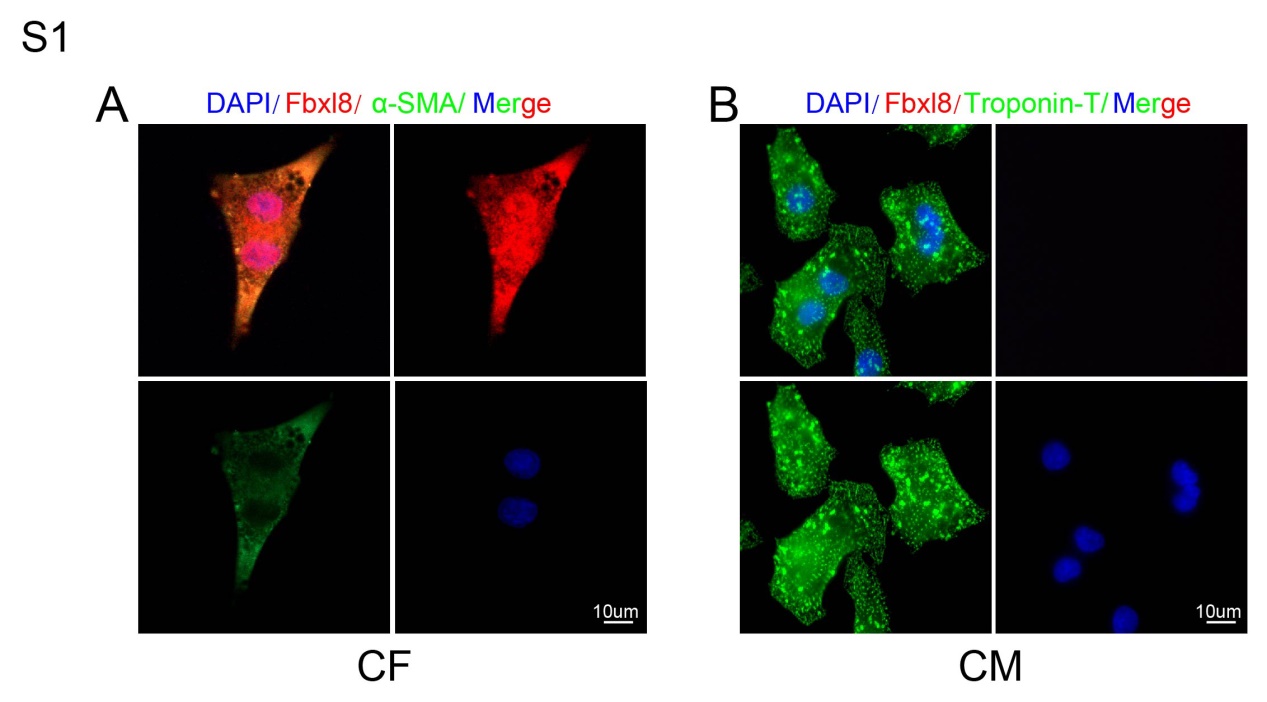
**

**Sfigure 1. FBXL8 is selectively enriched in CFs.** Representative immunofluorescence of FBXL8 expression in CFs (A) and CM (B).

**
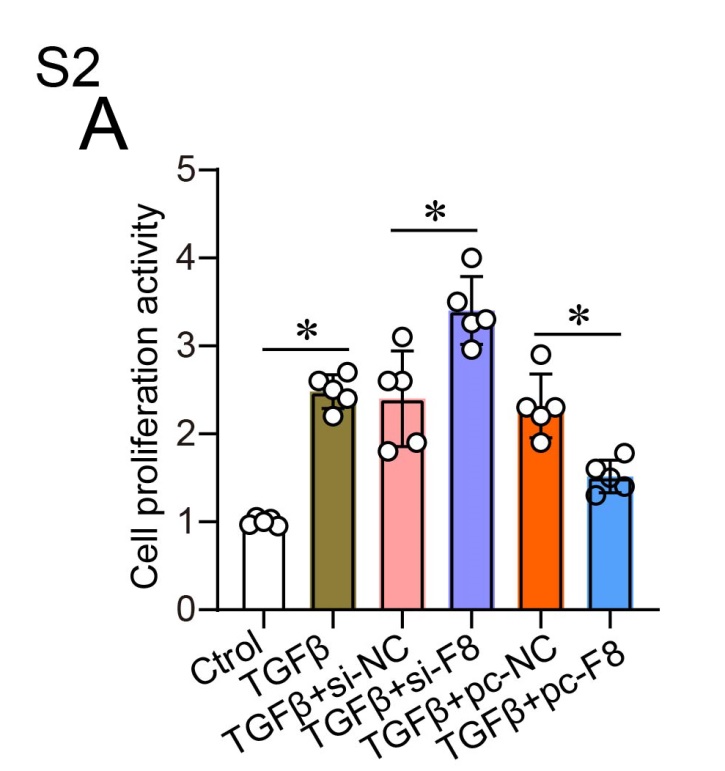
**

**Sfigure 2. FBXL8 negatively regulates TGF-β1-induced myofibroblast proliferation.** (A) The effects of FBXL8 on cell proliferation were determined by CCK8 assays, N=5.

**
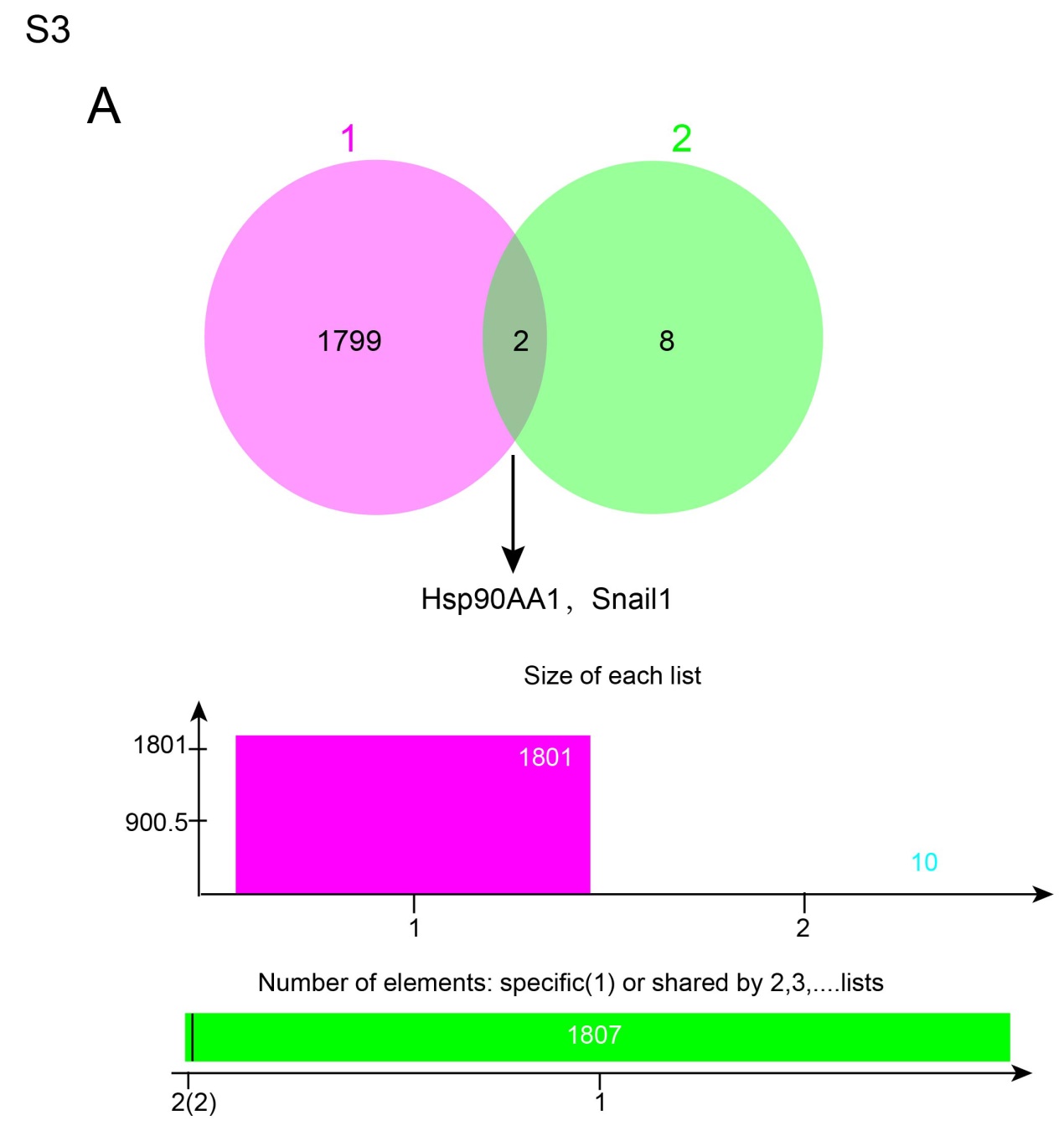
**

**Sfigure 3. The potential binding proteins of FBXL8 in database.** (A) Comparison of potential binding proteins of FBXL8 in the database of BioGRID with MI-related proteins in the database of DisGeNet.


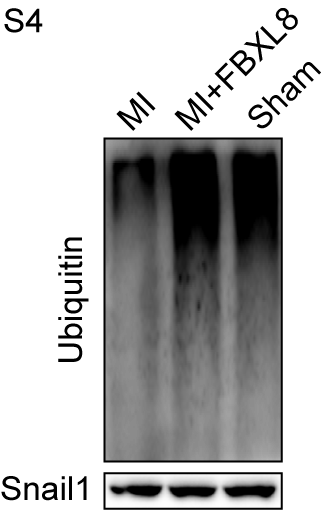


**Sfigure 4. FBXL8 increased the ubiquitination level of Snail1.**

**STable 1. Primers used in this study**

| **Name** | Sequence(5’- 3’) |
| --- | --- |
| **FBXL8** | Forward:AGAGGAAGTTTTGGGGCTCA  Reverse:ATTCCAGGCTCAGGTTGTGa |
| **Snail1** | Forward:AAGCCCAACTATAGCGAGCt  Reverse: TTTTGCCACTGTCCTCATCG |
| **GAPDH** | Forward:ATAGACAAGATGGTGAAGGTC  Reverse:TACTCCTTGGAGGCCATG TAG |
| **β-actin** | Forward:CGAGGCCCTCTGAACCCTA |
|  | Reverse:CAGCCTGCCTTTCTTTTGAC |
| **si-FBXL8** | GACTGCACCTTGCTAGTAT |
