# Supplementary figures and images for: FBXL8 inhibits post-myocardial infarction cardiac fibrosis by targeting Snail1 for ubiquitin-proteasome degradation
Source: Cell Death Dis. 2024 Apr 13;15(4):263. doi: 10.1038/s41419-024-06646-1 (PMC11016067; doi:10.1038/s41419-024-06646-1)

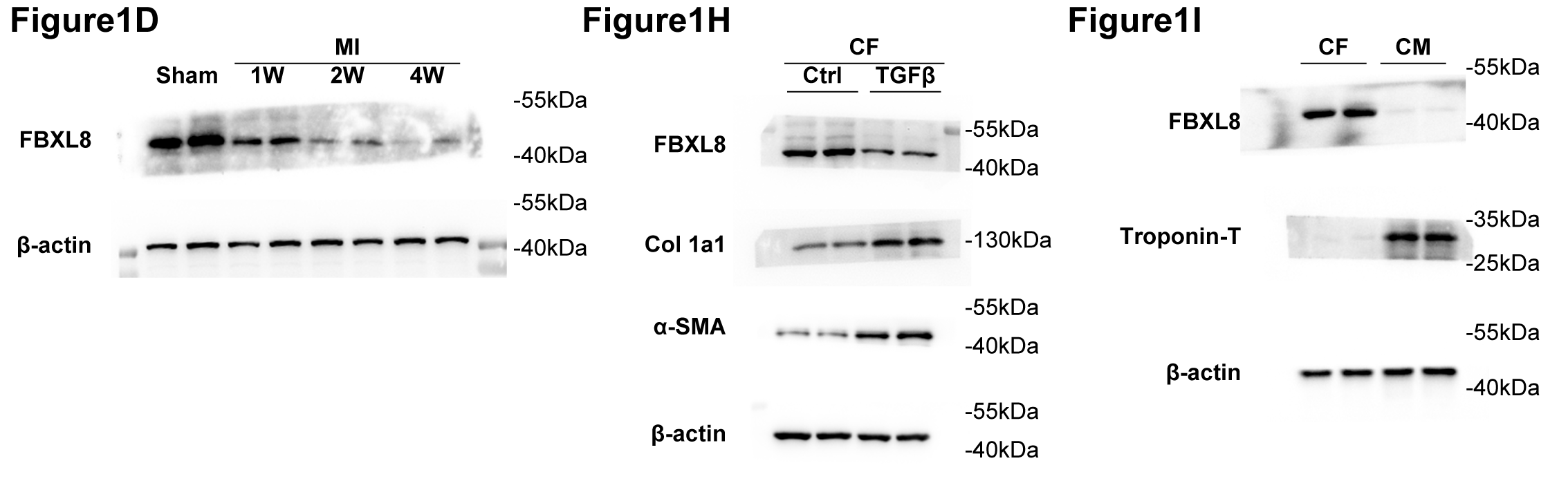


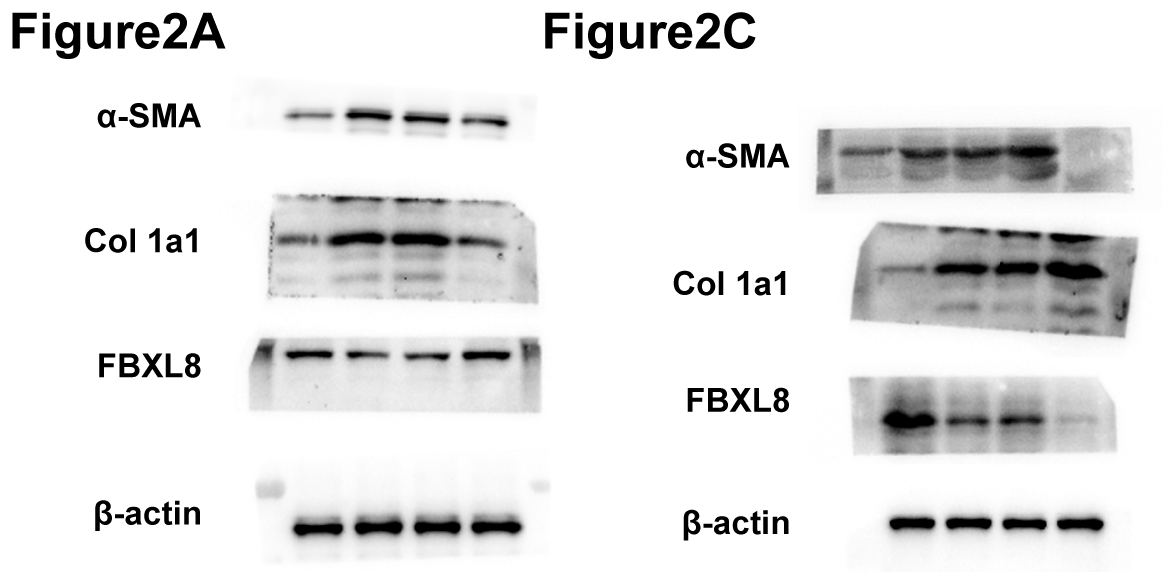


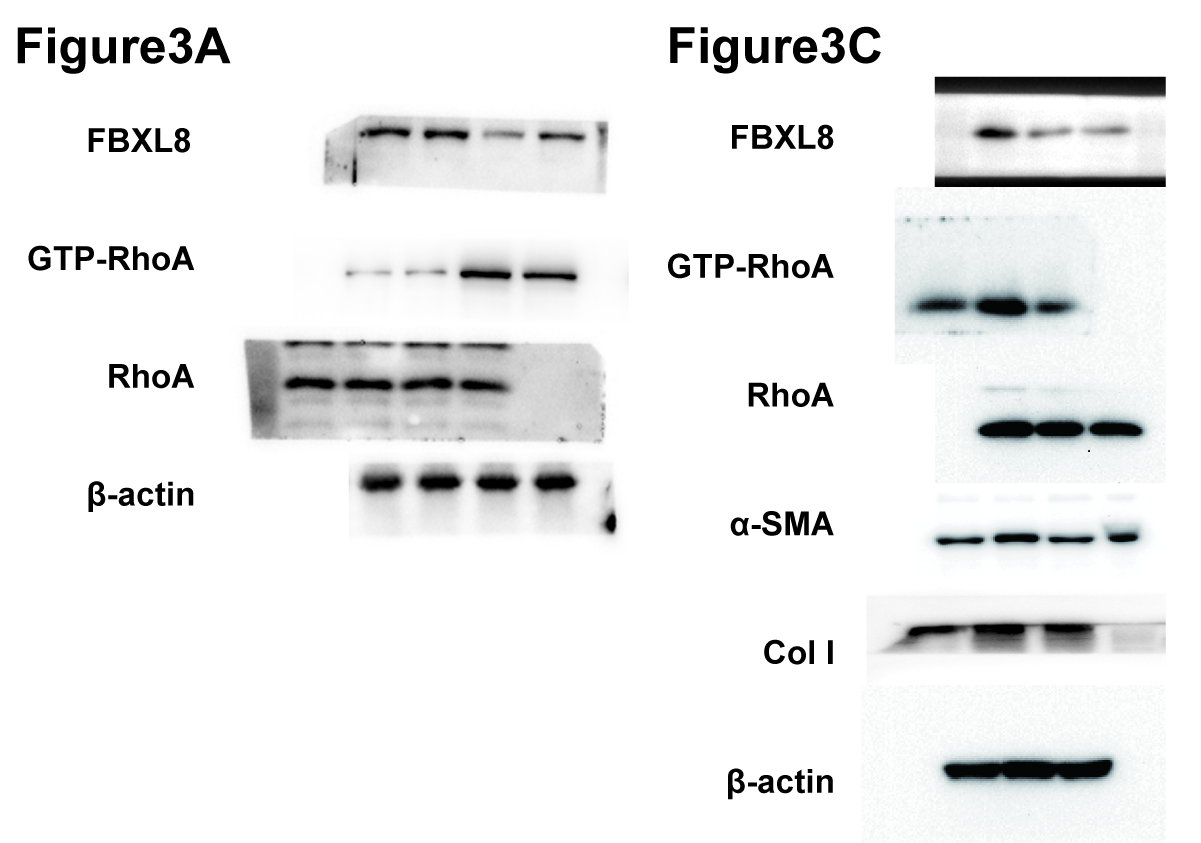


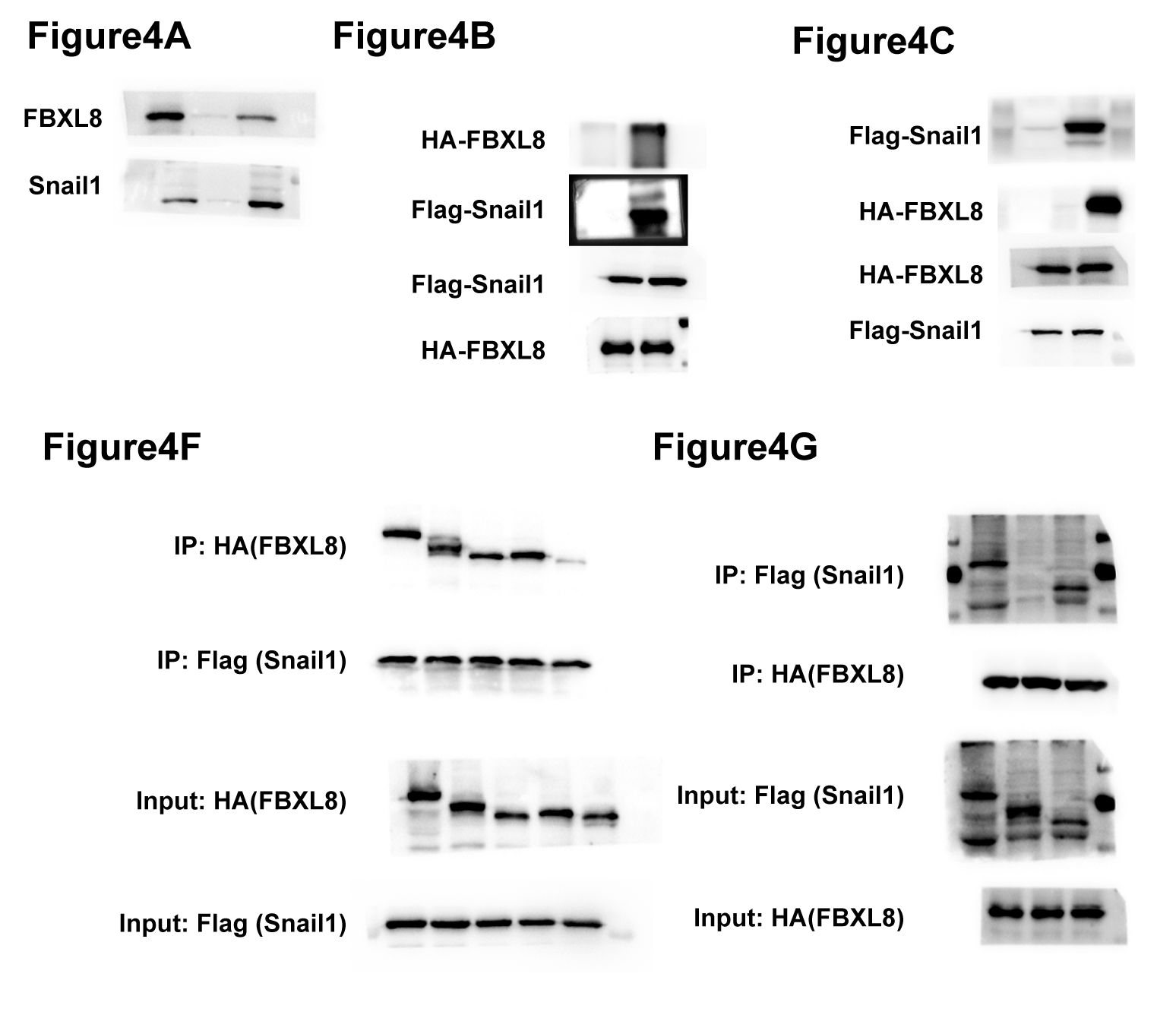


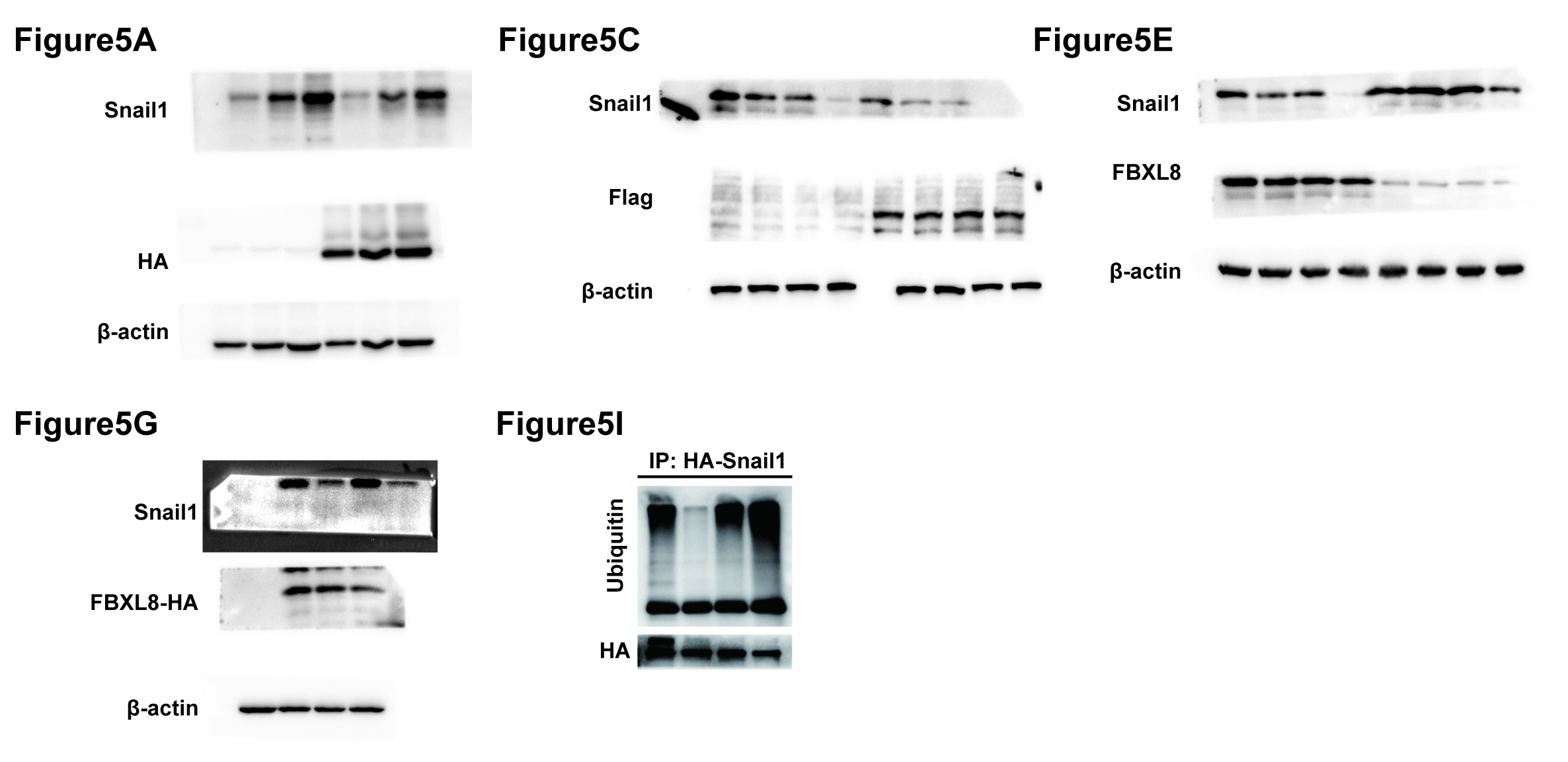


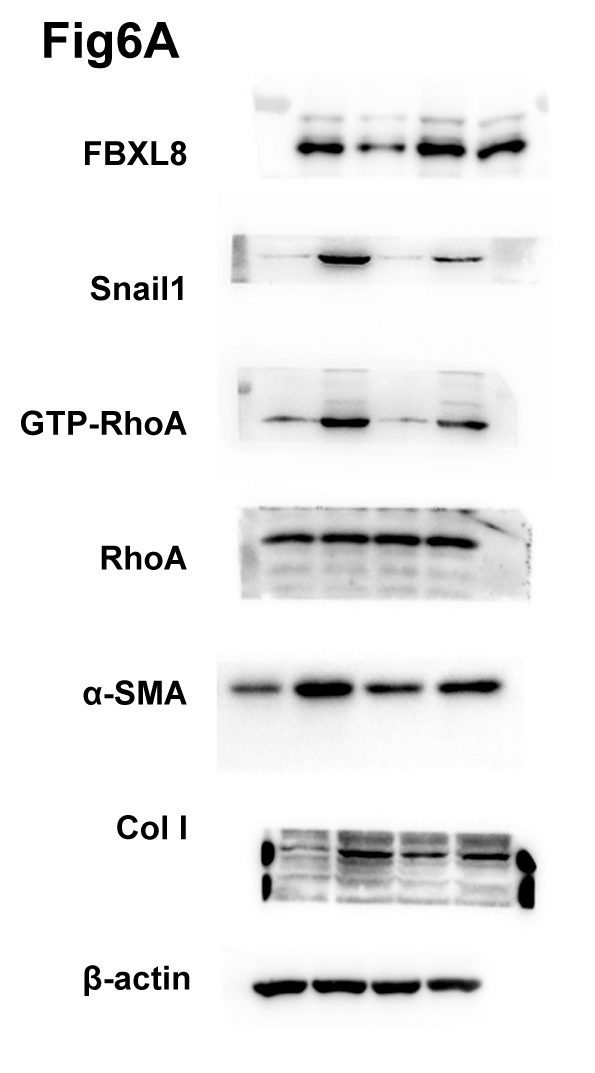


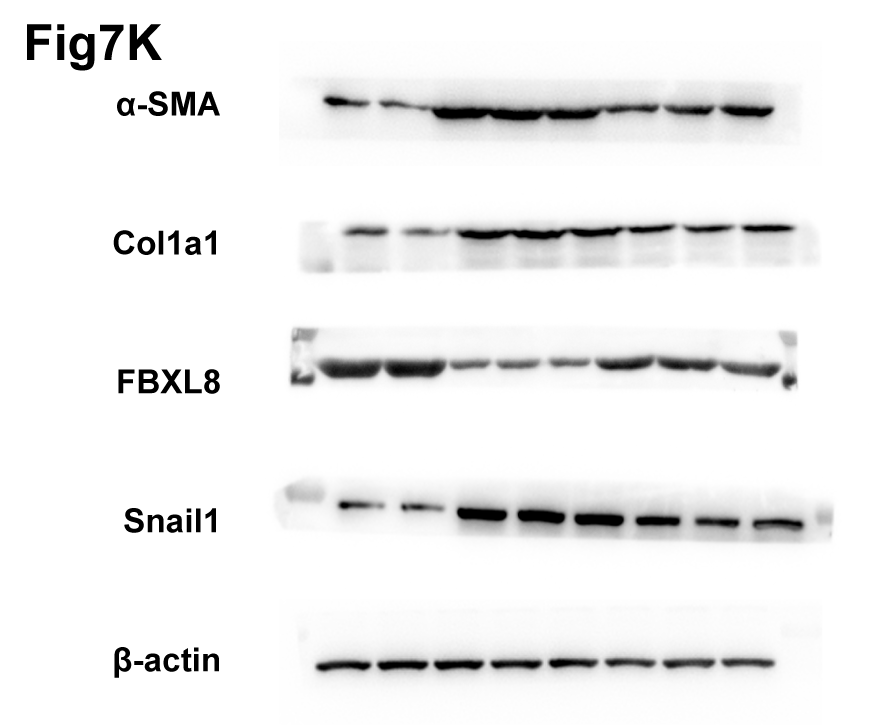

Supplement: Supplementary file 2 — Western blot original data [file 41419_2024_6646_MOESM2_ESM.docx]
